# Nutrition research in the first decade of 21^st^ century in Iran: the necessity of road Map

**DOI:** 10.1186/2193-1801-2-262

**Published:** 2013-06-13

**Authors:** Farnaz Khoshnevisan, Majid Hajifaraji, Nahid Salarkia, Azadeh Aminpour, Maryam Rassi, Nargess Abbassgholi, Payam Tarighi, Madjid Shakiba

**Affiliations:** National Nutrition and Food Technology Research Institute, Shahid Beheshti University of Medical Sciences (SBUMS), Tehran, Iran; National Nutrition and Food Technology Research Institute, Faculty of Nutrition Sciences and Food Technology, Shahid Beheshti University of Medical Sciences (SBUMS), 3# Baran, West Arghavan, Farahzadi Blvd, Shahrak Qods, P.O. Box: 19395-4741, Tehran, Iran; Nutrition and Food Policy Research Department, National Nutrition and Food Technology Research Institute, Shahid Beheshti University of Medical Sciences (SBUMS), 3# Baran, West Arghavan, Farahzadi Blvd, Shahrak Qods, P.O. Box: 19395-4741, Tehran, Iran; Department of community nutrition, Faculty of Nutrition Sciences and Food Technology, National Nutrition and Food Technology Research Institute, Shahid Beheshti University of Medical Sciences (SBUMS), Tehran, Iran; Library and Information Sciences, National Nutrition & Food Technology Research Institute, Shahid Beheshti University of Medical Sciences (SBUMS), Tehran, Iran; Medical Library and Information Sciences, National Nutrition & Food Technology Research Institute, Shahid Beheshti University of Medical Sciences (SBUMS), Tehran, Iran; Sina Trauma & Surgery Research Center, Tehran University of Medical Sciences (TUMS), Tehran, Iran; Advanced Diagnostic and Interventional Radiology Research Center, Tehran University of Medical Sciences (TUMS), Tehran, Iran

**Keywords:** Nutrition, Research type, Subject heading, Design, Policy

## Abstract

Due to important role of nutrition research in understanding of relevant health subjects and lack of periodic situation analysis of nutrition articles in Iran, this study was conducted to assess nutrition publications in two time intervals of 2001–2005 and 2006–2010 in Farsi scientific journals. A title to title search was performed in all medical, basic science, agricultural and veterinary journals in a 10-year period. All the article titles were placed in techniques, foods, nutritional biochemistry and physiology, nutrition and health, and clinical nutrition subject headings based on Nutrition Abstracts and Reviews Series A (NARA) database. The publication type and the study design were also determined. Statistical analysis was carried out by chi square to test temporal changes. There were 2127 Farsi publications. The original articles consisted 98.1% of the articles. Interventional and survey articles composed 28.1% and 20.8% of the publication types, respectively. Researchers were mostly interested in descriptive articles. Regarding subject, nutrition and health, and clinical nutrition were of the first and second time period interests, respectively. In comparison between the two time periods, regarding subject heading, the proportion of nutrition and health publications showed a significant decline; while, the proportion of clinical nutrition publication showed a remarkable rise. The publication type, subject and study design of the article do not follow coordinated planning and policy making. Therefore, these researches are not efficient enough to solve nutritional problems in our community properly. Planning of the research priorities in the field of food and nutrition with the agreement and participation of all stakeholders is a necessity.

## Introduction

There are many ways to assess scientific products and the process of research in different fields of which the number of articles published and the numbers of articles based on the type of publication are some of these simple and prevalent criteria (Sanson-Fisher et al. 2006; Habibi et al. 2010). Nutritional research plays an important role in our understanding of relevant health subjects which may be used in the prevention, control and treatment of diseases. Epidemiologic evaluation of articles to determine the type of publication and the design of the study are beneficial in the optimum utilization of these research results (Bittar et al. 2011).

The number and proportion of the articles published based on subject classification in a certain period of time demonstrates the propensity of researchers towards specific subjects which depends on many factors including the funding sector, the sponsor’s benefit, the government advisor and popular topics. These researchers are always after the latest results published in the articles of their field of interest and based on these results they promote their point of view (Sabate et al. 1999).

In Iran also scientific products have an upgrading process and many papers have been written in this concern (Habibi et al. 2010; Aminpour & Kabiri 2009; Eskrootchi et al. 2011; Malekzadeh et al. 2001; Moin et al. 2005; Osareh & Mardefat 2005). It is worth mentioning that the field of nutrition science among other fields of medical science, has been ignored and apart from Ensafi and Gharibi in Iran’s Book of Knowledge (Ensafi & Gharibi 2004), other authors have not mentioned anything about it. Besides, most of the Iranian authors were not pursuing the study design used in the articles and were only tracking the number of articles and the number of citations to these articles leading them away from assessment the article structure (Khoshnevisan et al. 2011). Therefore the authors assessed nutrition publications in Farsi scientific journals in two time periods of 2001–2005 and 2006–2010.

## Materials and methods

The study group consisted of all the articles published in Farsi scientific journals in Iran. All journals in fields related to nutrition subjects were chosen from Scientific Information Database (SID), Indexing Articles Published in Iran Biomedical Journals (IranMedex) and Iranian Magazines Database (Magiran). In order to recognize articles related to nutrition science and to prevent repetition, the name and number of all the scientific journals existing in Iran related to nutrition subjects were defined which were 249 Farsi journals in the field of nutrition, medicine, veterinary, basic science and agriculture. Then all the articles’ titles were read from all the Farsi journal issues from 2001–2010. In the next step, those titles related to nutrition were confirmed and saved their full texts under the journal’s published year. Finally the selected articles were categorized into five subject headings of Techniques, Foods, Nutritional Physiology and Biochemistry, Nutrition and Health, and Clinical Nutrition according to Nutrition Abstracts and Reviews Series A (NARA) database from the Center for Agricultural Bioscience International (CABI). NARA is a specialized database that covers subjects related to food and health from “food composition” and “food safety” to “obesity”, “parenteral nutrition” and “allergy”. The design of the study was divided into descriptive and analytical and the publication types were divided into original, review, systematic review with/without meta-analysis, brief report and letter to editor. The analytical articles were subcategorized as interventional, in vivo, in vitro, case–control and cohort (Kothari 2008). Also, the population type was determined. Subsequently the article was coded and entered to the PASW 18 program for analysis. Statistical analysis was carried out by Chi square for significance of the process between the two 5-year time periods.

## Results

**Figure 1:**
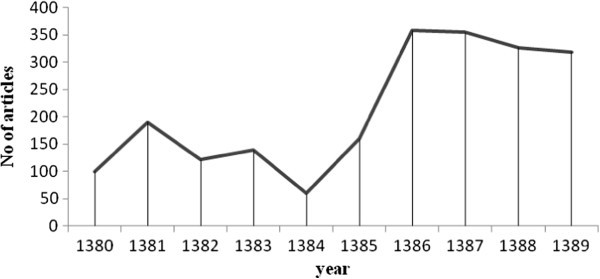
**Annual trend of the number of published Farsi nutrition articles from 2001 to 2010.**

There were 609 articles in the first five years (2001–2005) and 1518 in the second five years (2006–2010); an overall 2127 Farsi articles published in scientific journals throughout these ten years (2001–2010). As demonstrated in Figure 1, assessing the annual production of Farsi publications it is obvious that in the last 5 years there has been a notable rise.

Table 1 shows the publication types. No significant difference was observed between these two time periods regarding the type of publication.

**Table 1:** **The publication type of published Farsi nutrition articles in Iran, 2001-2010**

Publication type	Total	2001-2005	2006-2010	***Ρ among all groups***	***Ρ***^***1***^***For each group versus others***
		n (%)			
Total	2127 (100)	609 (28.6)	1518 (71.4)		
Original Research	2086 (98.1)	598 (98.2)	1488 (98)	0.72	0.8
Review	37 (1.7)	10 (1.6)	27 (1.79)		0.83
Systematic Review	1 (0.05)	-	1 (0.07)		0.99
Systematic Review and Meta analysis	1 (0.05)	-	1 (0.07)		0.99
Brief Report	1 (0.05)	-	1 (0.07)		0.99
Letter to editor	1 (0.05)	1 (0.2)	-		0.29
Total	2127 (100)	609 (100)	1518 (100)		

^*1*^Chi-square for linear trends.

Table 2 displays the designs of the studies. Assessing the design of Farsi articles, it may be concluded that clinical trials and interventions have had the highest importance among Iranian researchers with a higher proportion for surveys in the first time period (p<0.0001).

**Table 2:** **The study designs of Farsi nutrition articles published in Iran, 2001-2010**

Study design	Total	2001-2005	2006-2010	***Ρ among all groups***	***Ρ***^***1***^***For each group versus others***
		n (%)			
Total	2086 (100)	598 (28.7)	1488 (71.3)		
Clinical trials and interventions	455 (21.8)	91 (15.2)	364 (24.5)	<0.0001	<0.0001
Survey	433 (20.8)	171 (28.6)	262 (17.6)		<0.0001
Case–control	256 (12.3)	111 (18.6)	145 (9.7)		<0.0001
In vivo	243 (11.6)	79 (13.2)	164 (11)		0.16
In vitro	88 (4.2)	1 (0.2)	87 (5.9)		<0.0001
Cohort	48 (2.3)	10 (1.7)	38 (2.5)		0.23
Case study	12 (0.6)	5 (0.8)	7 (0.5)		0.34
Other descriptive	551 (26.4)	130 (21.7)	421 (28.3)		0.002
Total	2086 (100)	598 (100)	1488 (100)		

^*1*^Chi-square for linear trends.

The number of clinical trial articles performed on patients in hospitals were higher than community trial articles which were performed out of the hospital (73.6% versus 26.4% in the first time period and 63.7% versus 36.3% in the second time period).

Table 3 categorizes the articles regarding their subject. Comparing the subjects between the first and second time periods, a significant difference was found in all subject groups except for nutritional physiology and biochemistry.

**Table 3:** **Subject headings of Farsi nutrition articles published in Iran from 2001 to 2010**

Subject Headings	Total	2001-2005	2006-2010	***Ρ among all groups***	***Ρ***^***1***^***For each group versus others***
		*n* (%)			
Techniques	52 (2.5)	-	52 (3.4)	<0.0001	<0.0001
Foods	261 (12.3)	58 (9.6)	203 (13.4)		0.014
Nutritional physiology and biochemistry	354 (16.6)	87 (14.3)	267 (17.6)		0.064
Nutrition and Health	730 (34.3)	373 (61.2)	357 (23.5)		<0.0001
Clinical Nutrition	730 (34.3)	91 (14.9)	639 (42.1)		<0.0001
Total	2127 (100)	609 (100)	1518 (100)		

^*1*^Chi-square for linear trends.

**Figure 2:**
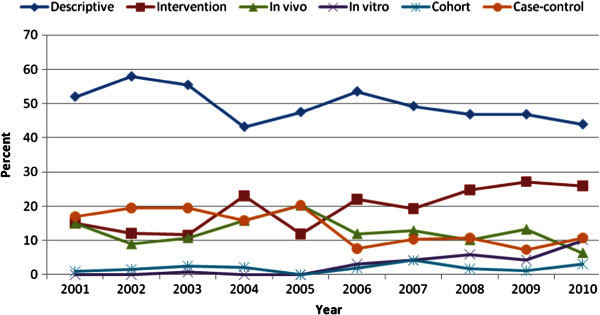
**The trend of study design in nutrition publications from 2001-2010.**

The trend of study design has been shown in Figure 2. Descriptive studies have always constituted the highest proportion of the study designs through these 10 years, regardless of its overall downward trend with a minimum percent in 2004 and a maximum in 2002.

**Figure 3:**
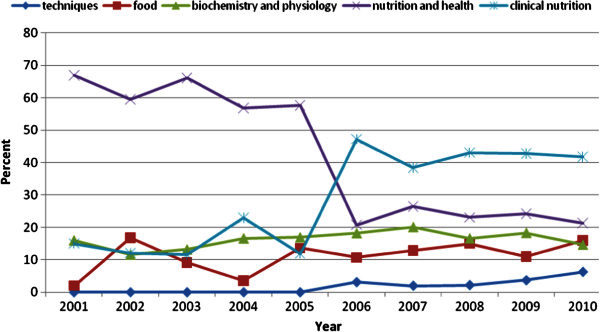
**The trend of subject heading in nutrition publications from 2001-2010.**

The nutrition and health subject shows a descending slope from 2001 to 2005, followed by a tremendous drop from 2005 to 2006; slightly rising afterwards. On the contrary, clinical nutrition has a peak at 2006; staying steady since then. Although food subject has had a decrease from 2002 to 2004 reaching its minimum number at 2004, it has managed to get to another peak at 2010 (Figure 3).

## Discussion

In this study, articles published in the last ten years have been assessed. The number of published articles in all fields has increased in the recent years in Iran (Habibi et al. 2010; Eskrootchi et al. 2011; Moin et al. 2005; Osareh & Mardefat 2005; Archumbault 2010; Osareh & Wilson 2002). The present study reveals that such an increase is also obvious in the field of nutrition which has been more conspicuous in the last 5 years (Figure 1). This development is attributed to the capability of human resource education, equipment and support by the authorities and scientific managers in research affairs and increase in the number of Farsi scientific journal published towards accomplishing the first to third 5-year plans and also more investment in science production (Moin et al. 2005). Despite this, the increase in scientific publications in the field of nutrition and food only indicates the quantitative increase of scientific activation in this field which is traditionally evaluated by the number of publications which has been answered by this study, but in order to evaluate scientific activity, attention should be paid towards other criteria such as used expenses in research and developing centers, the gross domestic expenditure for research and development to gross domestic product ratio, the number of employed staff in the scientific research department, the population under poverty, the per capita gross domestic product and the budget allocated for research. Many centers and research organizations spend their budget on researches in which new knowledge production and applicability are not the priorities and they develop publications and repeat other countries’ studies. Besides, with the day to day increase in the number of scientific journals in the field of medicine and the emphasis applied by the academic staff and researchers for publishing scientific publications, defining the quality of the published articles becomes an important issue (Habibi et al. 2010; Malekzadeh et al. 2001).

Most of the Farsi publications were original articles and the number of review articles was limited. These findings were congruent with studies performed by Abolghassemi Fakhree et al. and Habibi et al. (2010; Abolghassemi Fakhree & Jouyban 2011). In the review articles, only two systematic reviews were published in Farsi. Based on the fact that systematic reviews are one of the convenient tools for clinical guideline development and policy making in health issues and on the other hand as many of the policy makers, physicians and other medical professionals do not have enough time to update their information, systematic reviews are an appropriate way to reach this objective (Moher & Tricco 2008). It seems that researchers have not yet discovered the importance of these studies and that also policy makers and research planners do not have the enough understanding regarding the significance of review studies.

The researchers showed highest interest towards Farsi publications in clinical nutrition; consequently followed by health and nutrition, foods, nutritional physiology and biochemistry, and finally techniques. In other words, greater interest in research was devoted to the field of medicine (clinical nutrition) and disease control compared to other subjects such as health and nutrition which has the role of prevention and health promotion. The subject of foods in the last years, especially since 2007 has been beyond previously Farsi publications, which may be due to capacity building and having opportunity in the field of nutrition research. This publication rate increase may reflect the change in the consumers’ attitude and their expectation from food due to their insight to the probably positive effect of food in the prevention and control of diseases, the growing awareness towards the relation of food and physiological processes, improvement of food and technology and market progress in expanding new food products related to food research (Hilliam 2012; Kapsak et al. 2011; Stanton et al. 2001).

Ensafi and Gharibi’s study showed that Iranian nutrition publications made a small proportion in science production in the field of medicine (Ensafi & Gharibi 2004). Though, gradually the number of publications has increased in nutrition field owing it to the rise in non communicable diseases such as cardiovascular diseases, obesity and diabetes which are related to nutrition (Delavari et al. 2005; Nourbala & Mohammad 2001), the role of food in the control and treatment of diseases and community health (Kapsak et al. 2011) and the increase in the number of scientific journals.

Abolghassemi Fakhree et al., Habibi et al., Sanson-Fisher et al. and Bittar et al. have pointed to the fact that methodologically, descriptive studies have been the most used method by Iranian researchers and also studies performed worldwide (Sanson-Fisher et al. 2006; Habibi et al. 2010; Bittar et al. 2011; Abolghassemi Fakhree & Jouyban 2011). The reason is that the idea of such researches are more easily shaped in mind, the stages are performed more quickly and are published more conveniently. In addition, descriptive studies, which may be an analysis of a large data collection, with a higher applicability for the researcher in comparison to a study carried out on the community, are inexpensive and are more possible to reach the wrap-up. Although descriptive studies may for example give valuable information regarding the health pattern and its determining factors, they can not provide direct evidence about the state of change. Sanson-Fisher believed that a high number of descriptive studies is not ideal and research organizations should pay attention to these studies’ findings cautiously in policy making (Sanson-Fisher et al. 2006).

Clinical trial articles have shown an upgoing trend in Farsi journals. Habibi et al. evaluated the scientific products of Islamic countries and have detected that Iran has the second rank after Turkey in clinical trial articles. These types of publications have showed an increasing trend from 2007 to 2009 in Islamic countries (Habibi et al. 2010) which are compatible with our study. This growth may be attributed to the researchers’ interest and also the scientific journals’ authorities’ propensity towards publishing such articles. Based on the type of population (patients/community) in trial studies, it may be concluded that community trials carried out on community population have a lower number compared to clinical trials. Although trials are expensive and time-consuming, studying the community instead of the patient results in much higher time consumption and expense and also difficulty in controlling confounding factors. Furthermore, because health decision makers are mostly clinicians, emphasis is placed more on clinical trials (Review HaMRS 1998).

Case–control studies are relatively quick and inexpensive; consequently, leading to higher interest in researchers, but cohort studies are less favored due to being very time-consuming and expensive (Callas 2008).

In our study, the proportion of in vitro studies in nutrition has shown minor decrease in the second five-year period. Despite being second rank after Turkey, Iran is far beyond Turkey in this regard among other Islamic countries (Habibi et al. 2010).

Researchers have propensity towards problem solution instead of problem orientation. This change in direction is valuable only when a link is created between research priorities and researchers leading to choose an appropriate study design and publication type which is applicable when researchers and decision makers collaborate more efficiently. This is easily highlighted by Figure 3. The research map including research priorities is defined by various committed stakeholders in the food and nutrition field. The present study has illuminated the necessity of a protocol based on structure, nutrition database; leadership; participation of stakeholders; and issues related to food and nutrition. We have demonstrated the nutrition database as a reference for the above mentioned research map.

Assessing the trend of publication types, subjects and study designs, we concluded that the articles do not follow coordinated planning and policy making. However there are a few national research plans or policies mentioning some general priorities, the researchers do not follow them. One assumption could be the lack of authorities’ enforcement or special encouragements in related legislations. Furthermore, the articles are not efficient enough to solve nutritional problems in our community properly. Therefore putting a food and nutrition research map as our national program is a priority that should be focused on determinately.
